# Supplementary material for: Discovering SNP-disease relationships in genome-wide SNP data using an improved harmony search based on SNP locus and genetic inheritance patterns
Source: PLoS One. 2023 Oct 13;18(10):e0292266. doi: 10.1371/journal.pone.0292266 (PMC10575495; doi:10.1371/journal.pone.0292266)
Supplement: S1 Table — (DOCX) [file pone.0292266.s001.docx]

**S1 Table. Minimal dataset underlying Fig 2;** Power of the proposed algorithm (LDHS) for detecting disease-causing interactions) for Disease models with marginal effects (DMEs)

| **Fig1** | **Mean** | **S.D** | **S.E** | **# samples** |
| --- | --- | --- | --- | --- |
| **CSE** | 0.135 | 0.0795 | 0.0397 | 4 batches of 100 |
| **NHSA-DHSC** | 0.9075 | 0.0920 | 0.0460 | 4 batches of 100 |
| **EpiACO** | 0.09 | 0.0591 | 0.0295 | 4 batches of 100 |
| **MP-HS-DHSI** | 0.63 | 0.0935 | 0.0467 | 4 batches of 100 |
| **BEAM** | 0.015 | 0.0259 | 0.01295 | 4 batches of 100 |
| **Exhaustive Search** | 0.05 | 0.0316 | 0.0158 | 4 batches of 100 |
| **LDHS** | 0.97 | 0.0223 | 0.0111 | 4 batches of 100 |
