# Supplementary material for: Discovering SNP-disease relationships in genome-wide SNP data using an improved harmony search based on SNP locus and genetic inheritance patterns
Source: PLoS One. 2023 Oct 13;18(10):e0292266. doi: 10.1371/journal.pone.0292266 (PMC10575495; doi:10.1371/journal.pone.0292266)
Supplement: S2 Table — (DOCX) [file pone.0292266.s002.docx]

**S2 Table. Minimal dataset underlying Fig 3;** Power of the proposed algorithm (LDHS) for detecting interactions of different orders **for Disease models with no marginal effects (DNMEs)**

| **Fig2** | **Mean** | **S.D** | **S.E** | **# samples** |
| --- | --- | --- | --- | --- |
| **CSE** | 0.5675 | 0.1975 | 0.09875 | 4 batches of 100 |
| **NHSA-DHSC** | 0.3175 | 0.3544 | 0.1772 | 4 batches of 100 |
| **EpiACO** | 0.4 | 0.3537 | 0.1768 | 4 batches of 100 |
| **MP-HS-DHSI** | 0.83 | 0.1701 | 0.0850 | 4 batches of 100 |
| **BEAM** | 0.015 | 0.0259 | 0.0129 | 4 batches of 100 |
| **Exhaustive Search** | 1 | 0 | 0 | 4 batches of 100 |
| **LDHS** | 0.99 | 0.0173 | 0.0086 | 4 batches of 100 |
