# Supplementary material for: Discovering SNP-disease relationships in genome-wide SNP data using an improved harmony search based on SNP locus and genetic inheritance patterns
Source: PLoS One. 2023 Oct 13;18(10):e0292266. doi: 10.1371/journal.pone.0292266 (PMC10575495; doi:10.1371/journal.pone.0292266)
Supplement: Attachment — Submitted filename: Response to Reviewers.docx [file pone.0292266.s003.docx]

Dear Reviewers,

## Authors would like to express their gratitude for reviewing manuscript entitled “Discovering SNP-disease relationships in genome-wide SNP data using an improved harmony search based on SNP locus and genetic inheritance patterns”.

## The comments are addressed carefully and the answers are provided after each question (beginning with a ‘++’ mark). The most significant comment concerned the lack of detail regarding the algorithm. To address this comment, in addition to completing ‘section 3.2’ and adding the details of weighting and grouping method, the pseudocode of the proposed initialization is provided in Fig 1 of the revised manuscript.

## All changes are highlighted in “Revised Manuscript with Track Changes” file.

## Sincerely.

**Review Comments to the Author**

Please use the space provided to explain your answers to the questions above. You may also include additional comments for the author, including concerns about dual publication, research ethics, or publication ethics. (Please upload your review as an attachment if it exceeds 20,000 characters)

Reviewer #1: It appears that the paper successfully utilized harmony search in discovering SNP-disease relationships. However, it needs the following revisions:

= There are some more harmony search approaches applied in SNP. Please survey them also in literature section, such as:

- HS-MMGKG: A Fast Multi-objective Harmony Search Algorithm for Two-locus Model Detection in GWAS

- A Secure High-Order Gene Interaction Detecting Method for Infectious Diseases

++ Thank you. The previous work section is updated in the manuscript (lines 86-90).

= Section 2: Algorithm description is too short. Please provide algorithm pseudo-code and/or flow-chart.

++ Algorithm and flowchart are added (Fig 1 and Fig 2), also section “3.2 Weighting SNPs” is updated to add the details (lines 284-292).

Also, provide the difference among algorithms in terms of algorithm structures by refering [1]:

[1] Review of Harmony Search with Respect to Algorithm Structure,” Swarm and Evolutionary Computation, 48, 31-43, 2019.

++ Thank you. This is a very interesting paper to use and address for explaining what is done in the proposed method. The explanation is added and highlighted in “2 Materials and methods”. Corrections can be found in lines 132-145.

= Typos

- Table 1: In "HMCR = 0.98 0.98". Delete "= 0.98". Same for "Tau0=1, eta=1, alpha=1, beta=1"

++ corrected.

Reviewer #2: The authors propose an algorithm (Discovering SNP-disease relationships in genome-wide SNP data using an improved harmony search based on SNP locus and genetic inheritance patterns) that claims to integrate biological knowledge to improve harmony memory, however, this is not reflected in the code, including the main contribution in the authors' paper (identifying SNPs of different gene regions, grouping SNPs. However, it is not reflected in the code, including the main contributions in the authors' paper (identifying SNPs of different gene regions, grouping SNPs, and weighting SNPs) are also not specific enough.

++ Thank you. The description of the process is updated (highlighted in the manuscript, section 3.2 - lines 284-292), and also the pseudocode of the proposed method is provided in Fig 1.
